# Dietary *Lactobacillus plantarum* Supplementation Improves Growth Performance and Antioxidant Status and Upregulates Genes Related to the Toll/Imd and JAK-STAT Signaling Pathways in Red Claw Crayfish (*Cherax quadricarinatus*)

**DOI:** 10.3390/ani16071090

**Published:** 2026-04-02

**Authors:** Qin Zhang, Chongyang Du, Jiahao Zhao, Luoqing Li, Jianhang Ren, Tong Tong, Dapeng Wang, Rui Wang, Yongqiang Liu, Huizan Yang

**Affiliations:** 1 Guangxi Key Laboratory for Polysaccharide Materials and Modifications, Guangxi Marine Microbial Resources Industrialization Engineering Technology Research Center, School of Marine Sciences and Biotechnology, Guangxi Minzu University, 158 University Road, Nanning 530008, China; zhangqin@gxmzu.edu.cn (Q.Z.); duchongyang@stu.gxmzu.edu.cn (C.D.); zhaojiahao@stu.gxmzu.edu.cn (J.Z.); liluoqing@stu.gxmzu.edu.cn (L.L.); renjianhang@stu.gxmzu.edu.cn (J.R.); tongtong@gxmzu.edu.cn (T.T.); 2 Guangxi Key Laboratory for Aquatic Genetic Breeding and Healthy Aquaculture, Guangxi Academy of Fishery Sciences, 8 Qingshan Road, Nanning 530021, China; oucwdp@163.com (D.W.); raywongxx@163.com (R.W.)

**Keywords:** red claw crayfish, *Lactobacillus plantarum*, probiotic supplementation, hepatopancreas, innate immunity, crustacean aquaculture, signaling pathway, gene expression

## Abstract

This study conducted a 70-day feeding trial to evaluate the effects of dietary *Lactobacillus plantarum* supplementation (0, 0.1, 1.0, and 10.0 g/kg) in red claw crayfish (*Cherax quadricarinatus*). Growth performance, hepatopancreatic antioxidant-related parameters, and the expression of genes related to the Toll/Imd and JAK-STAT pathways were determined. Results showed that dietary *L. plantarum* supplementation significantly improved weight gain rate, specific growth rate, daily growth index, and feed efficiency, which were increased by 23.57%, 5.10%, 9.67%, and 7.90%, respectively, and reduced mortality rate by 60.00% compared with the control group (*p* < 0.05), enhanced the activities of glutathione peroxidase, catalase, superoxide dismutase and total antioxidant capacity, and reduced malondialdehyde levels in the hepatopancreas (*p* < 0.05). Furthermore, dietary *L. plantarum* supplementation was associated with the upregulated expression of key genes related to the Toll/Imd and JAK-STAT signaling pathways (*p* < 0.05). The expression levels of tumor necrosis factor-α, interleukin-1β, and transforming growth factor-β1 were also significantly increased (*p* < 0.05). Overall, these findings suggest that dietary *L. plantarum* supplementation improved growth performance and antioxidant-related and immune-related parameters in red claw crayfish, and that these effects were associated with the upregulated expression of genes involved in the Toll/Imd and JAK-STAT pathways. Among the tested treatments, 1.0 g/kg *L. plantarum* produced the most favorable overall response.

## Simple Summary

Red claw crayfish (*Cherax quadricarinatus*) is an important aquaculture species, but intensive farming may increase oxidative stress and impair immune-related functions. *Lactobacillus plantarum* is a widely used probiotic in aquaculture; however, its effects in red claw crayfish remain insufficiently understood. In this study, a 70-day feeding trial was conducted to evaluate the effects of dietary *L. plantarum* supplementation at 0, 0.1, 1.0, and 10.0 g/kg. The results showed that 1.0 g/kg *L. plantarum* produced the most favorable overall response, improving growth performance, antioxidant-related parameters, and the expression of several immune-related genes. These findings suggest that dietary *L. plantarum* may be beneficial for the healthy culture of red claw crayfish and provide a basis for its application in aquafeeds.

## 1. Introduction

Red claw crayfish (*Cherax quadricarinatus*) is a commercially important crustacean in China, and it has developed into a core aquaculture species by virtue of its fast growth rate, robust environmental adaptability, and superior meat quality [1]. With the increasing demand for high-quality aquatic products, the culture scale of red claw crayfish has expanded steadily, and intensive aquaculture has become a common farming mode. However, intensive aquaculture systems often face practical challenges, including water quality deterioration [2], nutritional imbalance in formulated feeds [3], and frequent disease outbreaks [4]. Such adverse factors can significantly compromise the growth performance and farming efficiency of red claw crayfish, thus posing substantial restrictions on the sustainable development of the crayfish farming industry.

To address these challenges, probiotics have been widely used in aquaculture as safe and environmentally friendly feed additives that help reduce antibiotic use and promote animal health [5]. Previous studies have shown that dietary supplementation with probiotics can exert beneficial effects by enhancing immune responses [6], promoting growth performance [7], improving water quality [8], and maintaining intestinal microbial homeostasis [9] in aquatic animals. *Lactobacillus plantarum*, a commonly used probiotic, has been reported to improve the growth and health status of aquatic animals through multiple mechanisms, including modulation of the gut microbiota, enhancement of immune-related responses, and promotion of nutrient absorption [10]. For instance, in common carp (*Cyprinus carpio*), dietary supplementation with *L. plantarum* significantly improved growth performance, digestive enzyme activities, and antioxidant immune parameters, while alleviating heat stress-induced damage [11]. Similarly, in white shrimp (*Litopenaeus vannamei*), *L. plantarum* supplementation activated immune-related gene expression, enhanced antioxidant and antibacterial capacities, and increased survival following *Vibrio alginolyticus* infection [12]. In addition, positive effects of *L. plantarum* on growth performance, immune responses, and antioxidant capacity have also been reported in blue swimming crab (*Portunus pelagicus*) [13], coho salmon (*Oncorhynchus kisutch*) [14], and Nile tilapia (*Oreochromis niloticus*) [15]. Nevertheless, research efforts addressing the utilization of *L. plantarum* in red claw crayfish aquaculture remain relatively scarce, and whether this probiotic is associated with changes in antioxidant-related and immune-related responses in this crustacean species remains to be clarified.

In the innate immune system of crustaceans, both the Toll/immune deficiency (Toll/Imd) and Janus kinase-signal transducer and activator of transcription (JAK-STAT) signaling pathways play crucial regulatory roles [16,17]. The Toll/Imd signaling pathway represents a central defense mechanism against pathogen infection. Specifically, the Toll pathway primarily recognizes pathogen-associated molecular patterns, such as lipopolysaccharides and peptidoglycans, thereby activating downstream nuclear factor-κB (NF-κB) transcription factors and inducing the expression of antimicrobial peptides to facilitate pathogen clearance [18]. In contrast, the Imd pathway mainly responds to peptidoglycan derived from Gram-negative bacteria, leading to the activation of downstream signaling molecules such as Relish and the subsequent induction of antimicrobial peptide expression [19]. Accumulating evidence indicates that activation of the Toll/Imd signaling pathway enhances immune capacity in crustaceans and improves their resistance to pathogen infection [20]. Meanwhile, the JAK-STAT signaling pathway, as an important intracellular signal transduction system, is widely involved in immune regulation as well as cell proliferation and differentiation [16]. This pathway is composed of cytokine receptors, JAK, and STAT, and has been reported to act synergistically with the Toll/Imd pathway in regulating immune responses in crustaceans [17]. Therefore, examining whether dietary probiotics are associated with changes in immune-related responses and the expression of genes related to these pathways in red claw crayfish remains of both theoretical and practical interest.

In summary, although *L. plantarum* has been widely applied in aquaculture, its effects on the growth performance, antioxidant status, and immune-related responses in red claw crayfish have not been systematically elucidated. In particular, information remains limited regarding its association with genes related to the Toll/Imd and JAK-STAT pathways in this species. Therefore, the present study aimed to investigate the effects of dietary *L. plantarum* supplementation on growth performance, hepatopancreatic antioxidant-related parameters, and immune-related gene expression in red claw crayfish. This study was intended to provide experimental evidence for the potential application of *L. plantarum* in red claw crayfish culture.

## 2. Materials and Methods

### 2.1. Diet Formulation

The freeze-dried *L. plantarum* strain (GXF5101) used in this study was obtained from a laboratory-maintained culture, with a viable cell concentration exceeding 10^10^ CFU/g [21].

For long-term storage at −80 °C, *L. plantarum* was cryopreserved in MRS (De Man, Rogosa & Sharpe) broth containing 30% (*v*/*v*) sterile glycerol, following the protocol outlined by Guo et al. [22]. Bacterial isolation was initiated by streaking the cryopreserved culture onto MRS agar, followed by anaerobic incubation at 37 °C for 48 h. From these plates, one isolated colony was randomly picked and aseptically transferred into liquid MRS medium. This inoculated medium was then subjected to aerobic incubation using an orbital shaker incubator set at 37 °C and 180 rpm for 24 h, allowing the culture to reach the logarithmic phase. Bacterial cells were subsequently collected by centrifugation at 4000× *g* for 10 min at 4 °C. The resulting cell pellet was washed three times using phosphate-buffered saline (PBS), transferred into a 50 mL centrifuge tube, and finally lyophilized for 48 h in a vacuum freeze dryer. The resulting freeze-dried powder contained an active bacterial count of at least 10^10^ CFU/g and was stored at −80 °C until further use in experiments.

Based on previous studies and safety evaluations [14], four different dietary treatments were designed with gradually increasing supplementation levels of *L. plantarum*. Specifically, the dietary treatments consisted of a control group (CK, without *L. plantarum* supplementation, 0 g/kg), a low-dose group (LL, 0.10 g/kg), a medium-dose group (ML, 1.00 g/kg), and a high-dose group (HL, 10.00 g/kg). The raw materials for the basal diet were purchased from Guangdong Hengxing Feed Industry Co., Ltd., Guangzhou, China. These raw materials were first crushed and sieved through a 60-mesh sieve, followed by uniform mixing with freeze-dried *L. plantarum* powder at the preset dosage levels, and finally subjected to pelleting. To maintain the quality of the diets, all formulated feeds were vacuum-dried until the moisture content dropped below 10%, then packaged and stored in a dark environment at −20 °C. Fresh feed batches were prepared every 4 d to reduce physicochemical degradation to a minimum and prevent microbial contamination. The proximate composition of the experimental diets formulated for red claw crayfish is summarized in Table 1.

In accordance with the method developed by Liu et al. [23], the viable count of *L. plantarum* in the experimental diets was determined by means of the plate counting method. In detail, 1 g of feed sample from each of the four groups was precisely weighed, homogenized thoroughly in 9 mL of sterilized 0.85% physiological saline with a vortex mixer, and underwent serial tenfold dilutions spanning from 10^−3^ to 10^−8^. Then, 100 μL aliquots of each diluted sample were aseptically plated onto the surfaces of sterilized MRS agar plates. For each dilution, three replicate plates were prepared to ensure statistical reliability. The inoculated plates were transferred into an anaerobic incubator maintained at 37 °C with a permissible deviation of ±0.5 °C, where they were incubated for 48 h. After this period, a quantitative evaluation of *L. plantarum* colonies, which displayed diameters ranging from 0.5 to 2 mm, was performed using a colony counter.

The viable cell count of *L. plantarum* was calculated with the following formula:

(1) Viable count (CFU/g) = B × (C/10) × f

wherein, *B* represents the total number of colonies grown on the MRS agar plates, *C* denotes the number of colonies identified as *L. plantarum* among ten randomly selected colonies, and *f* stands for the dilution factor.

According to the quantitative analysis performed using the MRS agar plate counting technique, no viable *L. plantarum* cells were identified in the feed samples from the control group (CK). For the experimental groups supplemented with *L. plantarum*, the viable bacterial counts were quantified as follows: the low-dose group (LL) exhibited 0.79 ± 0.12 × 10^6^ CFU/g, the medium-dose group (ML) showed 0.87 ± 0.18 × 10^7^ CFU/g, and the high-dose group (HL) reached 1.02 ± 0.07 × 10^8^ CFU/g.

### 2.2. Experimental Crayfish and Culture

The juvenile red claw crayfish employed in the present study were obtained from the Nanfan Breeding Base of the Guangxi Institute of Fisheries Science, Nanning, China. The initial mean body weight and body length were 0.13 ± 0.02 g and 0.58 ± 0.02 cm, respectively. To facilitate acclimation to the new environment and reduce transportation- and environment-induced stress, all individuals were acclimated for 7 d in a recirculating aquaculture system prior to the initiation of the feeding trial. After the acclimation period, the juveniles with uniform body size, intact morphology, healthy appearance, and robust vitality were selected to ensure comparability among experimental groups and the reliability of the experimental results. These selected juveniles were then used for the feeding trial.

The feeding experiment was carried out in culture tanks (1 × 2 × 1 m) with the water depth maintained at 70 cm. Each culture tank was fitted with 16 PVC pipes as hiding places, and continuous aeration was supplied through air stones. Water hyacinth (*Eichhornia crassipes*) was added to each tank to provide shelter and help maintain water quality and the overall culture environment, with the plants covering approximately 20–30% of the water surface area. Throughout the experimental period, the water temperature was kept at 26 ± 1 °C, while the dissolved oxygen concentration varied between 4.7 and 6.2 mg/L. The ammonia nitrogen concentration was maintained below 0.25 mg/L, nitrite-nitrogen below 0.01 mg/L, and nitrate-nitrogen was kept in the range of 0–10 mg/L. Additionally, the pH value was stabilized between 7.6 and 7.8, and an approximately natural local photoperiod of about 12 h light:12 h dark was maintained throughout the experiment.

A completely randomized design was adopted in this experiment, consisting of four dietary treatments with three replicates per treatment, resulting in a total of 12 culture tanks. Each tank was stocked with 50 young crayfish, with a total of 600 individuals involved in the 70-day feeding trial. Throughout the experimental period, the growth performance and mortality rate of crayfish in each tank were monitored, and the number of surviving individuals was recorded at regular intervals. The daily feeding amount was adjusted to approximately 5% of the total body weight of crayfish in each tank, and it was flexibly modified according to the feeding activity of the crayfish. The young crayfish were fed twice daily at 08:30 and 18:30. This feeding schedule was determined based on routine nursery management practice and preliminary observations of feeding activity in juvenile red claw crayfish. After the morning feeding, feces and residual feed were siphoned off, followed by partial water replacement. Approximately one-third of the total water volume in each tank was replaced during each water change to ensure the stability of water quality.

### 2.3. Sample Collection

Upon completion of the 70-day feeding trial, all red claw crayfish were subjected to 24 h of fasting. Subsequently, 12 crayfish individuals were randomly selected from each replicate for sample collection. The selected crayfish were rinsed thoroughly with sterile 0.85% physiological saline and anesthetized on ice prior to the performance of dissection. The final body mass of the crayfish was determined using a high-precision analytical balance (Sartorius CPA225D, Singapore, accuracy: ±0.01 g). Following this, the specimens were placed on a fully sterilized work surface and dissected under strict aseptic conditions. The hepatopancreas was quickly dissected out, promptly frozen in liquid nitrogen, and then stored at −80 °C for subsequent analysis. To determine the enzyme activities in the hepatopancreas, approximately 100 mg of hepatopancreatic tissue from each individual was homogenized in pre-cooled 0.85% physiological saline (pH 7.0) at a tissue-to-saline ratio of 1:9 (*w*/*v*), yielding a 10% (*w*/*v*) tissue homogenate. The homogenates were centrifuged at 1000× *g* for 10 min at 4 °C, and the resulting supernatants were harvested and used for the subsequent determination of enzyme activities.

### 2.4. Growth Performance Calculations

In accordance with the method established by Zhang et al. [24], the weight gain rate (*WGR*, %), specific growth rate (*SGR*, %/day), daily growth index (*DGI*, %/day), feed efficiency (*FE*, %) and mortality rate (*MR*, %) of red claw crayfish were calculated using the following formulas:

(2) $$\mathrm{WGR}\;\left(\%\right)\;=\;\frac{W_{1}\;-\;W_{0}}{W_{0}}\;\times \;100\%$$

(3) $$\mathrm{SGR}\;(\%/\mathrm{day})=\frac{\ln W_{1}-{\;\ln W}_{0}}{T}\;\times \;100\%$$

(4) $$\mathrm{DGI}\;\left(\%/\mathrm{day}\right)=\frac{{W_{1}}^{1/3}\;-\;{W_{0}}^{1/3}\;}{T}\;\times \;100\%$$

(5) $$\mathrm{FE}\;(\%)=\frac{W_{1}-W_{0}}{W_{S}}\;\times \;100\%$$

(6) $$\mathrm{MR}\left(\%\right)=(1-\frac{N_{1}}{N_{0}})\;\times \;100\%$$

where *W*_0_ and *W*_1_ represent the initial and final mean body weights (g), respectively; *W_S_* represents the total dry feed intake per tank (g); *T* represents the feeding duration (d); and *N*_0_ and *N*_1_ represent the initial and final numbers of crayfish per tank, respectively.

### 2.5. Determination of Antioxidant Enzyme Activities

The hepatopancreatic supernatants obtained following the method established by Zhang et al. [25] were employed to determine the activities of antioxidant-related enzymes. The activities of alkaline phosphatase (AKP) and acid phosphatase (ACP) were assayed via a microplate-based enzyme activity method. The activity unit of AKP was defined as the quantity of enzyme that generates 1 mg of phenol per gram of tissue protein upon incubation with the substrate at 37 °C for 15 min and was denoted as King units (KU/g prot). Correspondingly, the activity unit of ACP was defined as the quantity of enzyme that generates 1 mg of phenol per gram of tissue protein upon incubation with the substrate at 37 °C for 30 min, with the activity also expressed as King units (KU/g prot). Total antioxidant capacity (T-AOC) was measured using the ABTS radical cation scavenging assay, and one activity unit of T-AOC was defined as an absorbance (OD value) increase of 0.01 per milliliter of reaction solution per milligram of tissue protein per minute at 37 °C, denoted as mmol/mg prot. Superoxide dismutase (SOD) activity was determined by the WST-1 method, where one unit was defined as the amount of enzyme that induces a 50% inhibitory rate per milligram of tissue protein in 1 mL of reaction solution at 37 °C (U/mg prot). Catalase (CAT) activity was assayed via the ammonium molybdate-based colorimetric method, with one unit defined as the quantity of enzyme that degrades 1 μmol of H_2_O_2_ per milligram of tissue protein per second (U/mg prot). Malondialdehyde (MDA) content was determined using the thiobarbituric acid (TBA) colorimetric method and expressed as nmol/mg prot. Glutathione peroxidase (GSH-PX) activity was measured by a colorimetric assay, where one unit was defined as the amount of enzyme that causes a 1 mol/L reduction in glutathione (GSH) concentration per milligram of tissue protein per minute at 37 °C (U/mg prot). Glutathione (GSH) content was also determined via a colorimetric assay and denoted as nmol/mg prot. All detection kits were purchased from Nanjing Jiancheng Bioengineering Institute (Nanjing, China), and all experimental operations were conducted in strict accordance with the manufacturers’ operating instructions.

### 2.6. Determination of Relative Gene Expression

According to the method established by Zhang et al. [25], total RNA was isolated from hepatopancreatic samples using the MiniBEST Universal RNA Extraction Kit (TaKaRa, Dalian, China), with all experimental procedures strictly adhering to the manufacturer’s operating protocols. RNA integrity was assessed via 1% agarose gel electrophoresis, while RNA concentration was quantified with a NanoDrop spectrophotometer (Thermo Fisher Scientific, Waltham, MA, USA). Complementary DNA (cDNA) was generated using the PrimeScript™ RT Master Mix (Perfect Real Time; TaKaRa, Dalian, China). Quantitative real-time polymerase chain reaction (RT-qPCR) was carried out with TB Green^®^ Premix Ex Taq™ II (Tli RNaseH Plus; TaKaRa, Dalian, China) in a Q2000B Real-Time PCR System (LongGene, Hangzhou, China).

Gene-specific primers were designed for the selected target genes, including tumor necrosis factor receptor-associated factor 6 (*traf6*), *akirin*, interferon regulatory factor 4 (*irf4*), Toll-like receptor 6 (*tlr6*), Toll-like receptor 2 (*tlr2*), *imd*, tumor necrosis factor α (*tnf-α*), interleukin-1β (*il-1β*), transforming growth factor β1 (*tgf-β1*), *jak*, and *stat*. *β-actin* was employed as the housekeeping gene to normalize the relative transcriptional expression levels of the above target genes. In the present study, *β-actin* was selected as the reference gene based on its previous use in red claw crayfish and related crustacean RT-qPCR studies. Nevertheless, the use of a single reference gene should be considered a limitation of the present analysis. Melting curve analysis exhibited a single peak for all PCR amplicons, which demonstrated high amplification specificity and further validated the specificity of the designed primers. The detailed sequences of all primers are listed in Table 2.

### 2.7. Data Statistics and Analysis

All experimental data were first input into Microsoft Excel 2019 for preliminary preprocessing. Subsequent statistical analyses were performed with SPSS Statistics 25.0 software (IBM Corporation, Armonk, NY, USA). One-way ANOVA was used, followed by Tukey’s multiple comparisons test for post hoc analysis. All experimental results are expressed as the mean ± standard error (SE), and a *p*-value < 0.05 was set as the threshold for statistical significance. All experimental figures were constructed using GraphPad Prism 9 software (GraphPad Software Inc., San Diego, CA, USA). The relative transcriptional expression levels of genes detected by RT-qPCR were calculated via the 2^−ΔΔCt^ method [28], with the obtained results normalized against the expression level of the *β-actin* housekeeping gene.

## 3. Results

### 3.1. Effects of Lactobacillus plantarum Supplementation on Growth Performance in Red Claw Crayfish

**Figure 1:**
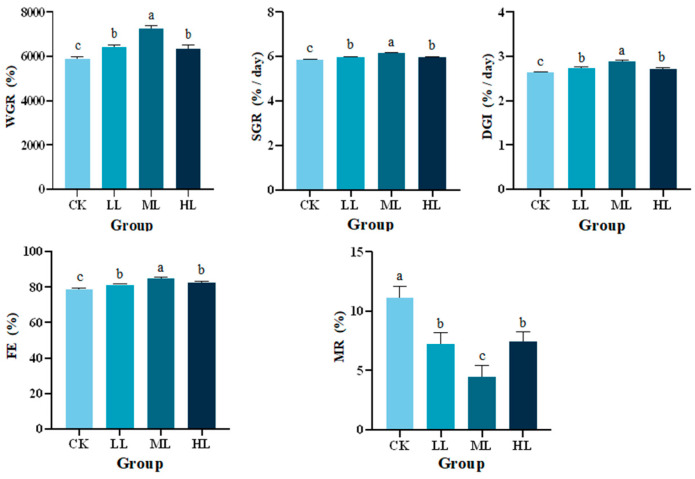
Effects of *Lactobacillus plantarum* supplementation on weight gain rate (WGR), specific growth rate (SGR), daily growth index (DGI), feed efficiency (FE), and mortality rate (MR) of red claw crayfish. Data are presented as mean ± SE (n = 3). Different superscript letters above bars indicate significant differences among treatments within the same parameter (*p* < 0.05). CK, control diet without *L. plantarum* supplementation; LL, diet supplemented with 0.10 g/kg *L. plantarum*; ML, diet supplemented with 1.00 g/kg *L. plantarum*; HL, diet supplemented with 10.00 g/kg *L. plantarum*.

As shown in Figure 1, following the 70-day feeding period, dietary supplementation with *L. plantarum* resulted in significant elevations in WGR, SGR, DGI, and FE in red claw crayfish relative to the control (CK) group (*p* < 0.05). For SGR, the LL, ML, and HL groups were all significantly higher than the CK group (adjusted *p* = 0.0032, *p* < 0.0001, and *p* = 0.0079, respectively). Similarly, FE was significantly increased in the LL, ML, and HL groups compared with the CK group (adjusted *p* = 0.0035, *p* < 0.0001, and *p* = 0.0086, respectively), and DGI was also significantly elevated in these groups (adjusted *p* = 0.0091, *p* < 0.0001, and *p* = 0.0007, respectively). Among all *L. plantarum*-treated groups, the ML group showed the most prominent enhancements in these parameters, with values significantly higher than those of the LL and HL groups (SGR: adjusted *p* = 0.0003 and 0.0002; FE: adjusted *p* = 0.0003 and 0.0001; DGI: adjusted *p* = 0.0024 and 0.0382, respectively), whereas no significant differences were observed between the LL and HL groups (SGR: adjusted *p* = 0.8797; FE: adjusted *p* = 0.8807; DGI: adjusted *p* = 0.2110). Moreover, the MR of all *L. plantarum*-supplemented groups was markedly decreased compared with the CK group (*p* < 0.05), and the ML group presented the lowest MR value, which was significantly lower than that of the other treated groups (*p* < 0.05).

### 3.2. Effects of Lactobacillus plantarum Supplementation on Antioxidant Enzyme Activities in Red Claw Crayfish

Data presented in Table 3 indicated that 70-day dietary administration of *L. plantarum* led to marked elevations in GSH-PX, GSH, CAT, SOD, and T-AOC contents in the hepatopancreas of red claw crayfish, relative to the control (CK) group (*p* < 0.05). Among all *L. plantarum*-supplemented treatments, the ML group showed the most remarkable increments in GSH-PX, GSH, and CAT levels, which were notably higher than those detected in other supplemented groups (*p* < 0.05).

In comparison with the CK group, ACP activity was significantly elevated in both ML and HL groups (*p* < 0.05), whereas no significant difference was found between the LL group and the CK group (*p* > 0.05). AKP activity was significantly increased only in the ML group (*p* < 0.05), with no statistically significant differences observed among the LL, HL, and CK groups (*p* > 0.05).

In addition, MDA content in the hepatopancreas was significantly decreased in all *L. plantarum*-treated groups compared with the CK group (*p* < 0.05). The ML group exhibited the lowest MDA level, which was significantly lower relative to the other supplemented groups (*p* < 0.05).

### 3.3. Effects of Lactobacillus plantarum Supplementation on the Toll/Imd and JAK-STAT Signaling Pathway in Red Claw Crayfish

**Figure 2:**
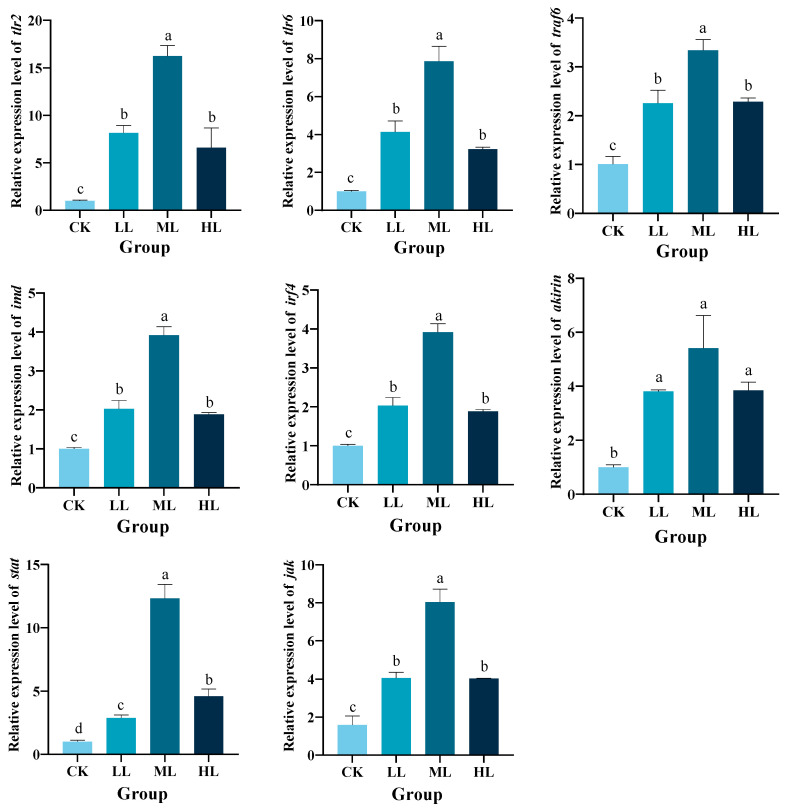
The effects of *Lactobacillus plantarum* supplementation on the relative expression levels of toll-like receptor 2 (*tlr2*), toll-like receptor 6 (*tlr6*), tumor necrosis factor receptor-associated factor 6 (*traf6*), immune deficiency homolog (*imd*), *akirin*, regulatory factor 4 (*irf4*), janus kinase (*jak*), and signal transducer activator of transcription (*stat*) genes in the hepatopancreas of red claw crayfish. All the data are presented as mean ± SE (n = 3). Within the same figure, different superscript letters indicate significant differences between the values (*p* < 0.05). CK, control diet without *L. plantarum* supplementation; LL, diet supplemented with 0.10 g/kg *L. plantarum*; ML, diet supplemented with 1.00 g/kg *L. plantarum*; HL, diet supplemented with 10.00 g/kg *L. plantarum*.

As shown in Figure 2, dietary supplementation with *L. plantarum* for 70 d significantly upregulated the expression of key genes involved in the Toll/Imd signaling pathway compared to the CK group (*p* < 0.05), including *traf6*, *akirin*, *imd*, *irf4*, *tlr6*, and *tlr2* in the hepatopancreas of red claw crayfish. Among the supplemented groups, the *traf6*, *akirin*, *imd*, *irf4*, *tlr6*, and *tlr2* genes in the ML group exhibited the highest expression levels and were significantly higher than those in the other *L. plantarum* supplemented groups (*p* < 0.05).

As shown in Figure 2, dietary supplementation with *L. plantarum* for 70 d significantly upregulated the expression of key genes involved in the JAK/STAT signaling pathway compared to the CK group (*p* < 0.05), including *jak* and *stat* in the hepatopancreas of red claw crayfish. Among the supplemented groups, the *jak* and *stat* genes in the ML group exhibited the highest expression levels and were significantly higher than those in the other *L. plantarum*-supplemented groups (*p* < 0.05).

### 3.4. Effects of Lactobacillus plantarum Supplementation on the Expression of Genes Related to Inflammatory Response in Red Claw Crayfish

**Figure 3:**
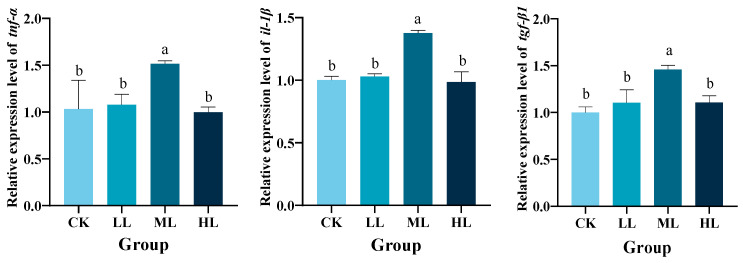
The effects of *Lactobacillus plantarum* supplementation on the relative expression levels of tumor necrosis factor α (*tnf-α*), interleukin 1β (*il-1β*), and transforming growth factor β1 (*tgf-β1*) genes in the hepatopancreas of red claw crayfish. All the data are presented as mean ± SE (n = 3). Within the same figure, different superscript letters indicate significant differences between the values (*p* < 0.05). CK, control diet without *L. plantarum* supplementation; LL, diet supplemented with 0.10 g/kg *L. plantarum*; ML, diet supplemented with 1.00 g/kg *L. plantarum*; HL, diet supplemented with 10.00 g/kg *L. plantarum*.

As shown in Figure 3, dietary supplementation with *L. plantarum* for 70 d significantly upregulated the expression of *tnf-α*, *il-1β*, and *tgf-β1* genes in the hepatopancreas of red claw crayfish in the ML group compared to the CK group (*p* < 0.05). However, no significant differences were observed among the CK, LL, and HL groups (*p* > 0.05).

## 4. Discussion

In aquaculture, growth performance and mortality rate are pivotal indicators of farming efficiency and sustainability [29]. In the present study, dietary supplementation with 1.0 g/kg *L. plantarum* significantly increased WGR, SGR, and DGI in juvenile red claw crayfish, indicating a growth-promoting effect. The improvement in FE suggests that *L. plantarum* may enhance nutrient utilization efficiency. In addition, MR was significantly reduced in all supplemented groups, suggesting that dietary *L. plantarum* may have improved the physiological status of red claw crayfish. However, this result should not be interpreted as direct evidence of enhanced disease resistance, because no immune challenge or pathogen infection assay was performed in the present study. These effects may be related to improved intestinal barrier function, modulation of gut microbiota homeostasis, and enhanced nutrient utilization [30]. *L. plantarum* has been reported to promote the localization and expression of tight junction proteins (e.g., occludin and ZO-1), thereby reducing intestinal permeability and inhibiting the colonization and invasion of pathogenic microorganisms. Improved intestinal barrier integrity may reduce immune burden and energy expenditure, thereby allowing more energy to be allocated to growth [31]. In addition, *L. plantarum* can modulate gut microbiota homeostasis to optimize intestinal microecology and epithelial function, thereby improving the intestinal digestive and absorptive environment and indirectly enhancing feed utilization and host growth [32]. Previous studies have shown that various antimicrobial substances are produced during *L. plantarum* metabolism, including bacteriocins and lactic acid, can inhibit the proliferation of pathogenic bacteria [33]. Moreover, short-chain fatty acids (SCFAs) generated by *L. plantarum* not only serve as an important energy source for intestinal epithelial cells [34] but also lower luminal pH and suppress the growth of harmful bacteria, further stabilizing the intestinal microenvironment [35]. Among these, butyrate has been reported to promote epithelial cell proliferation and differentiation and to exert immunomodulatory effects, thereby contributing to the maintenance of intestinal barrier integrity [36]. Collectively, these mechanisms may synergistically enhance nutrient absorption, ultimately leading to concurrent improvements in FE and growth performance. Consistent with this interpretation, previous studies have demonstrated that dietary supplementation with *L. plantarum* significantly promotes growth performance and feed efficiency in other aquatic animals, such as coho salmon [14], common carp [37], and white shrimp [38], further supporting the considerable application potential of *L. plantarum* in crustacean aquaculture. It should also be noted that the proximate composition of the experimental diets was broadly comparable among treatments (Table 1), suggesting that the observed differences were unlikely to be explained solely by major variations in basal nutrient composition. Nevertheless, because dietary *L. plantarum* supplementation was introduced through feed formulation, the immune-related responses observed in the present study should still be interpreted within the overall dietary context rather than as completely nutrition-independent effects.

However, dietary supplementation with *L. plantarum* at an optimal dosage improved the growth performance of red claw crayfish, whereas increasing the supplementation level to 10.0 g/kg did not yield further growth improvement and instead resulted in reduced performance. This finding suggests that the effects of *L. plantarum* in red claw crayfish were dose-dependent rather than linearly proportional to the supplementation level. Rather than increasing continuously with the supplementation dose, the efficacy of *L. plantarum* appears to be constrained within an optimal supplementation range, which may vary with the probiotic strain, host species, and rearing conditions [7,32]. Several non-exclusive hypotheses may explain the weaker response observed at 10.0 g/kg, although these possibilities were not directly tested in the present study. One possible explanation is that excessive supplementation of *L. plantarum* may have altered intestinal microbial homeostasis, thereby affecting intestinal function and reducing digestive and absorptive efficiency [39,40]. Another possible explanation is that a high probiotic dose may have increased the energy demand associated with physiological regulation, thereby reducing the energy available for somatic growth and other processes [41,42]. It is also possible that excessive accumulation of metabolic products, such as lactic acid and other organic acids, affected the intestinal environment and thereby influenced nutrient utilization efficiency [43]. Therefore, although dietary *L. plantarum* supplementation improved growth performance at an appropriate level, the weaker response at the higher inclusion level should be interpreted with caution. In the present study, 1.0 g/kg was identified as the most favorable supplementation level, whereas the weaker response at higher inclusion levels requires further verification.

Antioxidant capacity serves as a critical factor in maintaining physiological homeostasis and growth performance in red claw crayfish [44]. Antioxidant biomarkers, such as the activities of SOD, CAT, and GSH-PX, together with the contents of GSH, T-AOC, and MDA, are frequently used to evaluate antioxidant status and oxidative damage in aquatic organisms. Meanwhile, ACP and AKP are generally regarded as important biomarkers reflecting the innate immune status of crustaceans [45]. In the current trial, dietary administration of *L. plantarum* elevated the activities of SOD, CAT, and GSH-PX, together with the contents of GSH and T-AOC in the hepatopancreatic tissues of red claw crayfish, and significantly reduced MDA accumulation. Furthermore, the activities of innate immune-related enzymes including ACP and AKP, were also enhanced. These results suggest that dietary *L. plantarum* supplementation may enhance antioxidant capacity and improve basal immune status in red claw crayfish. The underlying mechanisms may involve two aspects. On the one hand, *L. plantarum* can synthesize bioactive metabolites that directly or indirectly enhance host antioxidant capacity [46]. Previous studies have shown that *L. plantarum* can synthesize antioxidant molecules de novo or facilitate the host’s synthesis and uptake of antioxidant-related substances and precursors. For example, it can synthesize antioxidant enzymes such as SOD, GR, and GSH-PX and promote the accumulation of GSH [47,48]. Additionally, *L. plantarum* can metabolize phenolic acids, such as hydroxycinnamic acids [49], and the fermentation process may release cell wall-bound phenolic compounds, thereby further enhancing antioxidant capacity [50]. Collectively, these findings suggest that *L. plantarum* may participate in the regulation of host antioxidant status through its metabolic activities. On the other hand, SCFAs produced by *L. plantarum* may regulate host redox balance by enhancing the expression and activities of antioxidant enzyme-related genes, thereby alleviating oxidative stress and maintaining redox homeostasis [51,52]. The enhancement of ACP and AKP activities may also be linked to the regulatory effects of *L. plantarum* on gut microecology, pathogen reduction, and immune burden alleviation. Similar findings have been reported in other studies: in white shrimp, supplementation with lactic acid bacteria significantly increased multiple nonspecific immune parameters, including ACP and AKP, suggesting an immunostimulatory effect of lactic acid bacteria-based probiotics on basal immunity in crustaceans [53]. In red claw crayfish, dietary supplementation with *Bacillus subtilis* has also been shown to enhance AKP and ACP activities, accompanied by increased resistance to infection [54]. In summary, dietary *L. plantarum* may help red claw crayfish maintain physiological homeostasis and improve antioxidant-related and nonspecific immune-related parameters, which may contribute to adaptation to environmental stress.

Findings from the current experiment revealed that dietary inclusion of *L. plantarum* upregulated the expression of core genes associated with the Toll/Imd and JAK-STAT signaling pathways in the hepatopancreas of red claw crayfish. Among all treatment groups, the 1.0 g/kg *L. plantarum* supplementation group displayed the most prominent upregulation of these target genes. These results suggest that dietary *L. plantarum* not only improved several immune-related parameters at the physiological and enzymatic levels but was also associated with the upregulation of genes related to innate immune signaling pathways at the molecular level.

Within the Toll/Imd signaling pathway, the upregulation of key genes, including *tlr2*, *tlr6*, *traf6*, and *imd*, suggests that dietary *L. plantarum* was associated with enhanced transcriptional responses related to immune recognition and signal transduction. Meanwhile, the upregulation of *akirin* and *irf4* suggests that the effects of dietary *L. plantarum* on the Toll/Imd pathway may extend beyond upstream receptor recognition and signal transduction to downstream transcriptional regulation. Akirin, as a key nuclear regulatory factor, functions as a molecular link between the Toll/Imd signaling pathway and NF-κB-dependent immune gene expression in invertebrates. Meanwhile, IRF-family transcription factors regulate the transcription of multiple immune-related genes in crustacean immune responses, participating in the amplification and integration of innate immune signaling [55,56]. Therefore, the upregulation of *akirin* and *irf4* may be associated with reinforced downstream immune effector responses within the Toll/Imd pathway. However, these transcriptional results alone are insufficient to confirm functional activation of the pathway or a direct enhancement of innate immune capacity. Unlike acute immune stress, the upregulation of Toll/Imd pathway-related genes observed in the present study was not accompanied by growth suppression or reduced survival. Instead, it occurred concurrently with significant improvements in WGR, SGR, and DGI, which is consistent with previous findings in crustaceans such as white shrimp, where probiotic supplementation has been shown to promote growth performance and immune function [57]. Previous studies have indicated that, in crustaceans, moderate immunoregulatory responses generally do not exert negative effects on growth, whereas excessive or acute immune stress may increase metabolic burden because of the high energy demands of immune defense [58]. Hernroth et al. [59], using Norway lobster (*Nephrops norvegicus*) as a model species, reported that under simulated environmental stress conditions, alterations in immune function were accompanied by adjustments in energy allocation, indicating a trade-off between immune responses and energy metabolism. Excessive immune activation may therefore occur at the expense of growth and other physiological processes. Therefore, the transcriptional and enzymatic responses observed in the present study may reflect a mild and sustained immunoregulatory state. However, because no pathogen challenge or functional validation was performed, these findings should not be interpreted as direct evidence of enhanced host defense. This balance is conducive to the maintenance of growth performance and is consistent with the current understanding of the coordinated regulation between immune modulation and metabolic homeostasis in crustaceans [58].

In addition to the Toll/Imd pathway, the present study also found that the expression of the key JAK-STAT signaling pathway genes *jak* and *stat* was upregulated, with the most pronounced effects observed in the 1.0 g/kg group, suggesting that dietary *L. plantarum* was associated with the upregulation of genes related to the JAK-STAT pathway. As an important component of the innate immune system in crustaceans, the JAK-STAT signaling pathway plays a role in mediating cytokine signal transduction, regulating the transcription of immune-related genes, and maintaining immune homeostasis [60]. Previous studies have indicated that in crustaceans, the JAK-STAT pathway usually acts in coordination with the Toll/Imd pathway, participating in the integration and amplification of immune signaling following pathogen recognition [61]. In studies on Chinese white shrimp (*Fenneropenaeus chinensis*), viral infection has been shown to upregulate the expression of *jak*, *stat*, and their associated genes in immune-related tissues such as hemocytes and the hepatopancreas [62], indicating that the JAK-STAT pathway may be involved at an early stage of innate immune responses and participates in immune signal transduction following pathogen invasion. In addition, at the mechanistic level, crustaceans can couple pathogen recognition signals to the JAK-STAT pathway through specific pattern recognition molecules. For example, receptors such as Domeless can mediate the JAK-STAT signaling axis, thereby regulating the expression of downstream immune effector genes [63]. Taken together, evidence from both expression changes and molecular mechanisms indicates that the JAK-STAT pathway plays a conserved and regulatory role in pathogen defense in crustaceans.

Considering the expression changes in the inflammatory cytokines *tnf-α* and *il-1β*, as well as the immunoregulatory factor *tgf-β1*, observed in the present study, the effects of dietary *L. plantarum* are better interpreted as being associated with the upregulation of genes related to the JAK-STAT pathway rather than as direct evidence of functional activation [64]. Moderate upregulation of *tnf-α* and *il-1β* may indicate an enhanced immune-alert state; however, depending on the biological context, it may also reflect inflammatory stimulation. As cytokine-like molecules, they participate in the regulation of hemocyte proliferation and the production of antimicrobial effector molecules. Together, these transcriptional changes may contribute to host defense, but direct functional validation was not performed [65]. In contrast, TGF-β1, as an important immunoregulatory factor, may participate in limiting excessive inflammatory responses and maintaining immune homeostasis; however, the current study did not include functional assays to confirm this role [66,67]. In this context, the transcriptional responses of the JAK-STAT pathway are more likely not to simply reflect enhanced pro-inflammatory responses but rather may function as a regulatory link between inflammatory signaling and immunoregulatory responses, while direct functional confirmation is still lacking. In this process, the Toll/Imd pathway likely serves as an upstream input for immune signaling, whereas the JAK-STAT pathway may participate in regulating the intensity and duration of downstream immune responses, although this has not been verified experimentally. Together, these pathways may form a coordinated immunoregulatory network, while the precise functional interactions remain to be experimentally validated.

The potential mechanisms by which *L. plantarum* mediates the coordinated regulation of multiple immune pathways may involve two aspects. First, as a typical Gram-positive bacterium, microbe-associated molecular patterns (MAMPs) on the surface of *L. plantarum*, such as lipoteichoic acids and peptidoglycan, can be recognized by host pattern recognition receptors, thereby initiating upstream immune signaling pathways such as Toll/Imd and inducing downstream changes in the expression of inflammatory cytokines and immunoregulatory factors [68]. Second, the intestinal colonization and metabolic activity of *L. plantarum* may modulate host innate immune responses and immune homeostasis by altering local microecological structure, nutritional metabolic status, and the production of immune-related signaling molecules, thereby shaping the host immune signaling network [69,70].

Several limitations of the present study should be noted. Although the experimental diets showed broadly comparable proximate composition, probiotic-associated effects could not be completely separated from all dietary background influences. In addition, no pathogen challenge, protein-level validation, or other functional analyses were performed; therefore, the observed changes in enzyme activities and gene expression should be interpreted as evidence of immunomodulation rather than direct proof of enhanced disease resistance or functional activation of the Toll/Imd and JAK-STAT pathways. The study also did not assess whether the administered *L. plantarum* strain could establish or persist in red claw crayfish. Finally, all analyses were based on three replicates per treatment, and RT-qPCR normalization relied on a single housekeeping gene (*β-actin*). Future studies should incorporate pathogen challenge experiments, functional validation, assessment of strain persistence, and larger biological replication to further strengthen the mechanistic interpretation.

Overall, dietary *L. plantarum* supplementation improved growth performance, antioxidant-related parameters, and several immune-related indicators in red claw crayfish, with 1.0 g/kg producing the most favorable overall response. These responses were accompanied by the upregulation of genes related to the Toll/Imd and JAK-STAT pathways, suggesting that these pathways may be involved in the response to dietary *L. plantarum*.

## 5. Conclusions

In summary, dietary *L. plantarum* supplementation improved growth performance, antioxidant-related parameters, and the activities of several nonspecific immune-related enzymes in red claw crayfish. These changes were accompanied by the upregulation of genes related to the Toll/Imd and JAK-STAT pathways, suggesting that these pathways may be involved in the response to dietary *L. plantarum* supplementation. Among the tested supplementation levels, 1.0 g/kg produced the most favorable overall response. The present study provides experimental support for the use of *L. plantarum* as a dietary additive in red claw crayfish culture, although further functional validation is needed to clarify the underlying mechanisms.

## Figures and Tables

**Table 1:** The proximate composition of the experimental diets formulated for red claw crayfish (g/kg of dry diet).

Ingredients	Groups			
	CK	LL	ML	HL
*Lactobacillus plantarum*	0	0.10	1.00	10.00
Fish meal	485.00	485.00	485.00	485.00
Soybean meal	119.30	119.20	118.30	109.30
Sorghum flour	106.50	106.50	106.50	106.50
Wheat flour	139.00	139.00	139.00	139.00
Corn flour	45.00	45.00	45.00	45.00
Soy Lecithin	10.00	10.00	10.00	10.00
Fish oil	15.20	15.20	15.20	15.20
Gelatin	20.00	20.00	20.00	20.00
Calcium carbonate	10.00	10.00	10.00	10.00
Choline chloride	5.00	5.00	5.00	5.00
Mineral premixes ^a^	20.00	20.00	20.00	20.00
Vitamin premixes ^b^	20.00	20.00	20.00	20.00
Vitamin C	5.00	5.00	5.00	5.00
Proximal composition (% Dry Matter)				
Dry material	92.56	92.56	92.56	92.56
Ash	7.76	7.76	7.76	7.76
Ethereal extract	7.40	7.40	7.40	7.40
Crude protein	35.20	35.20	35.20	35.20
Crude lipid	7.84	7.84	7.84	7.84
Fiber	3.43	3.43	3.43	3.43

Note: ^a^ Mineral premixes (mg/kg): KCl, 0.5; MgSO_4_·7H_2_O, 0.5; ZnSO_4_·7H_2_O, 0.09; MnCl_2_·4H_2_O, 0.0234; CuSO_4_·5H_2_O, 0.005; KI, 0.005; CoCl_2_·2H_2_O, 0.0025; Na_2_HPO_4_, 2.37. ^b^ Vitamin premixes (mg/kg): Vitamin B_12_, 0.02; Vitamin A acetate, 5000 IU; Vitamin D_3_, 4000 IU; α-tocopherol acetate, 100 IU; menadione, 5; thiamine HCl, 60; riboflavin, 25; pyridoxine HCl, 50; folic acid, 10; dl-capantothenic acid, 75; nicotinic acid, 5; biotin, 1; inositol, 5.

**Table 2:** Primer sequences for RT-qPCR in red claw crayfish.

Gene	Primer Sequence (5′→3′)	Amplicon Size (bp)	Tm (°C)	Gene Bank
*β-actin* ^1^	F: CGCCTGTCCGCTGGAATAAT	135	60	XM_053800817.1
	R: ACGATGGAAGGGAAGACAGC			
*traf6* ^2^	F: GTGCCACAGTCCACCATTCT	262	60	XM_053772658.1
	R: TACCTCTGGCCGCATGAAAG			
*akirin* ^3^	F: ACGCCGCAAGATATTACAGTGTGG	112	60	XM_053784413.1
	R: TGATGGTGAGGTAGGACAGACAGG			
*imd* ^4^	F: CATACCTCCCCGTCTGTGTCA	[20]	60	[20]
	R: CCATCTAACCCACCTGCTGTC			
*irf4* ^5^	F: CAGCGAAGTGTTCCGAGTTCCC	[26]	60	[26]
	R: TATGCCTCCTCCCGTGTGTTCTC			
*tlr6* ^6^	F: CTACAGTGCCAATGATGCTACCTAC	105	60	XM_053797426.1
	R: TCGCTGAAGTCTCTGGAGTGAAG			
*tlr2* ^7^	F: CTCGGACAAGGAGCGGTTAGTTTC	131	60	XM_053771523.1
	R: TTCTGATTGATAACCTGCTGGAGTCTG			
*tnf-α* ^8^	F: ACAGCATTAGTGAGAGCAGCAATC	123	60	XM_053772658.1
	R: CATTAGGACACATAACTGGTCTGAGG			
*il-1β* ^9^	F: ACGGTCACAGCCTCTAATGGTAC	78	60	XM_053781109.1
	R: CTCTCGGTAGTTCGGATTGGTTTG			
*tgf-β1* ^10^	F: CTCCAACACCACCTGAAGATAGATTG	98	60	XM_053797306.1
	R: AGTAACAGTGACATAGCAGTAACCATC			
*jak* ^11^	F: TGTGAGGCATAACAGTAACGAAGG	[27]	60	[27]
	R: GCCCAAGGAACTCAATGGAATG			
*stat* ^12^	F: CAGAAAATGTAGCCCACAGCCAG	[27]	60	[27]
	R: TAAAGCAAGGGGATTATTATTCAGG			

Note: F: Forward primer. R: Reverse primer. ^1^ *β-actin*: Non-regulatory internal reference gene; ^2^ *traf6*: Tumor necrosis factor receptor-associated factor 6 gene; ^3^ *akirin*: Akirin gene; ^4^ *imd*: Immune deficiency gene; ^5^ *irf4*: Interferon regulatory factor 4 gene; ^6^ *tlr6*: Toll-like receptor 6 gene; ^7^ *tlr2*: Toll-like receptor 2 gene; ^8^ *tnf-α*: Tumor necrosis factor α gene; ^9^ *il-1β*: Interleukin 1βgene; ^10^ *tgf-β1*: Transforming growth factor β1 gene; ^11^ *jak*: Janus kinase gene; ^12^ *stat*: Signal transducer and activator of transcription gene.

**Table 3:** The effects of *Lactobacillus plantarum* supplementation on the antioxidant enzyme activities in the hepatopancreas of red claw crayfish.

Index	Groups			
	CK	LL	ML	HL
GSH-PX ^1^ (U/mg prot)	574.01 ± 13.75 ^c^	660.65 ± 12.88 ^b^	746.52 ± 16.12 ^a^	664.15 ± 9.55 ^b^
GSH ^2^ (nmol/mg prot)	15.61 ± 1.75 ^c^	23.12 ± 0.14 ^b^	29.91 ± 0.39 ^a^	22.14 ± 1.05 ^b^
ACP ^3^ (KU/g prot)	39.07 ± 2.47 ^b^	52.34 ± 6.12 ^ab^	63.21 ± 4.49 ^a^	65.42 ± 6.47 ^a^
AKP ^4^ (KU/g prot)	18.99 ± 1.01 ^b^	20.7 ± 1.00 ^b^	29.15 ± 1.14 ^a^	21.35 ± 1.52 ^b^
CAT ^5^ (U/mg prot)	8.73 ± 0.33 ^c^	10.26 ± 0.41 ^b^	13.28 ± 0.28 ^a^	10.64 ± 0.70 ^b^
SOD ^6^ (U/mg prot)	46.74 ± 1.06 ^b^	54.96 ± 3.12 ^a^	55.03 ± 1.48 ^a^	53.27 ± 2.81 ^a^
T-AOC ^7^ (mmol/mg prot)	0.13 ± 0.01 ^c^	0.20 ± 0.01 ^b^	0.25 ± 0.01 ^a^	0.21 ± 0.03 ^ab^
MDA ^8^ (nmol/mg prot)	1.5 ± 0.01 ^a^	1.08 ± 0.01 ^c^	0.85 ± 0.01 ^d^	1.22 ± 0.07 ^b^

Note: All the data are presented as mean ± SE (n = 3). Within the same row, different superscript letters indicate significant differences between the values (*p* < 0.05). ^1^ GSH-PX: Glutathione peroxidase; ^2^ GSH: Glutathione; ^3^ ACP: Acid phosphatase; ^4^ AKP: Alkaline phosphatase; ^5^ CAT: Catalase; ^6^ SOD: Superoxide dismutase; ^7^ T-AOC: Total antioxidant capacity; ^8^ MDA: Malondialdehyde.

## Data Availability

The data that support the findings of this study are available from the corresponding authors upon reasonable request.
